# Parametric Optimization of Laser Additive Manufacturing of Inconel 625 Using Taguchi Method and Grey Relational Analysis

**DOI:** 10.1155/2020/9176509

**Published:** 2020-06-12

**Authors:** Bo Yang, Youbin Lai, Xiang Yue, Dongyang Wang, Yuhui Zhao

**Affiliations:** ^1^College of Engineering, Shenyang Agricultural University, Shenyang 110866, China; ^2^Shenyang Institute of Automation Chinese Academy of Sciences, Shenyang 110016, China

## Abstract

In order to improve the forming quality of the Inconel 625 cladding layer and make it to be more widely used. This paper addresses an experimental investigation on the influence of major process parameters like laser power, scanning speed, powder feed rate, and overlapping rate along with their interactions on surface roughness and width error of laser additive manufacturing process for forming Inconel 625 samples. Taguchi method and grey relational analysis were used to optimize the selected parameters, and the verification tests were carried out. The change of microhardness and microstructure in the overlapping zone and nonoverlapping zone of the cladding layer were studied by microhardness tester and scanning electron microscopy (SEM). The results show that the most significant effect in processing parameters on surface roughness and width error are both overlapping rate, and the optimal levels of laser power, scanning speed, powder feeder rate, and overlapping rate are 1800 W, 8 mm/s, 10 g/min, and 30%, respectively. Analysis of microstructure and composition showed that the content of Cr was high both in the Laves phase and matrix, the content of Nb in the Laves phase increased significantly and reached up to 24.48 wt%, and the Laves in the nonoverlapping zone was more compact than the overlapping zone.

## 1. Introduction

Inconel 625 is a solid-solution strengthened Ni-Cr-Mo alloy with a face-centered cubic structure [1]. Because of its excellent mechanical strength and resistance to creep and corrosion in harsh environments, Inconel 625 has been widely used in aerospace, chemical, petrochemical, and marine applications. However, many of the Inconel 625 components are highly complex shapes that are very expensive to produce due to extensive machining [2–6].

Laser additive manufacturing (LAM) is a new technology, which is widely used in aerospace, medical, military, and many other fields [7]. It integrates the latest achievements in the field of mechanical engineering, CAD/CAM, CNC or robot technology, laser technology, and materials science. And it can quickly transform the design idea into a prototype with a certain structure and function or manufacture parts directly, reducing the processing costs significantly [8–10]. In the LAM process, lots of different parameters such as laser power, scanning speed, and powder feed rate can be coupled together to influence the quality of the parts. Hence, optimizing the process parameters to obtain a quality part becomes a focus problem for many experts [11–18].

Nevertheless, most scholars only study the influence of multiple parameters on a single response target. Few experts have studied the influence of multiple parameters on multiple response targets in laser additive manufacturing of Inconel 625 alloy. In addition, the overlapping rate is a parameter that has an important influence on forming quality. The area of the overlapping zone will be increased when the overlapping rate is too high, the microstructure and microhardness of the cladding layer will be affected. However, the surface of the cladding layer will be uneven when the overlapping rate is too small. But many scholars often neglect its influence when they study the optimization of process parameters. In this paper, the comprehensive influence of parameters of laser power, scanning speed, power feed rate, and overlapping rate on the surface roughness and width error of the LAM process of Inconel 625 has been studied. And the microstructure and microhardness of overlapping zone and nonoverlapping zone have been explored. The Taguchi method and grey relational analysis have been used to analyse the influence of multiple parameters on multiple response targets, and the LAM parameters have been optimized.

## 2. Experimental Method

### 2.1. Experimental Equipment and Material

**Figure 1:**
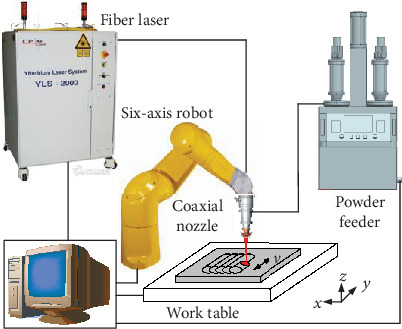
Schematic diagram of LAM.

The experiments were carried out with the LAM setup shown in Figure 1. The setup system includes a fiber laser with a 2000 W maximum output power, a robot with six-axis, a powder feeder system, and other auxiliary equipment. The base material used in the experiment was Inconel 625 alloy, and the Inconel 625 alloy was selected as alloy powder; the chemical composition of the powder was shown in Table 1. Before the experiment, the substrate was polished to remove the surface oxidation layer and improve its surface finish. Then, the substrate was cleaned with acetone to remove the oil on the surface. The Inconel 625 alloy powder was placed at 120°C vacuum environment for drying treatment.

### 2.2. The Taguchi Method

The Taguchi method is an optimized design technology founded by Genichi Taguchi PhD, a Japanese quality engineer. The method is mainly used for technology development, product development, and process development. The Taguchi method is divided into three procedures [19]: (1) Select factors and their levels according to experimental analysis; in this paper, laser power, scanning speed, powder feeding speed, and overlapping speed are selected as experimental factors, each of which are three levels. Factors and levels are shown in Table 2. Other factors are kept at their fixed level as mentioned in Table 2. (2) Select orthogonal array (OA) to conduct the experiments, the L_9_3^4^ Taguchi OA is selected to perform experiments. (3) Calculate the signal to noise (S/N) ratio and statistical analysis of variance (ANOVA), and then obtain the optimal process parameters.

### 2.3. DLF Experiments and Measurements

**Figure 2:**
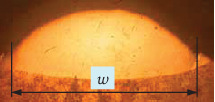
Geometry of section of single-track formation.

**Figure 3:**
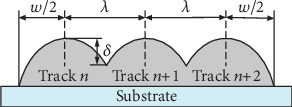
Schematic diagram of cross section of multitrack formation.

In the LAM experiment, the overlapping rate (OR) was computed by the following equation [20]:

(1) $$\begin{array}{c} \mu =\frac{w-\lambda }{w}, \end{array}$$

where *w* is the width of a single-track formation as shown in Figure 2, and *λ* is the offset distance between adjacent tracks as shown in Figure 3.

In the experiment, the overlapping rate is decided by the offset distance between adjacent tracks. According to Eq. (1), the distance between adjacent tracks can be expressed as follows:

(2) $$\begin{array}{c} \lambda =\left(1-\mu \right)\cdot w. \end{array}$$

**Figure 4:**
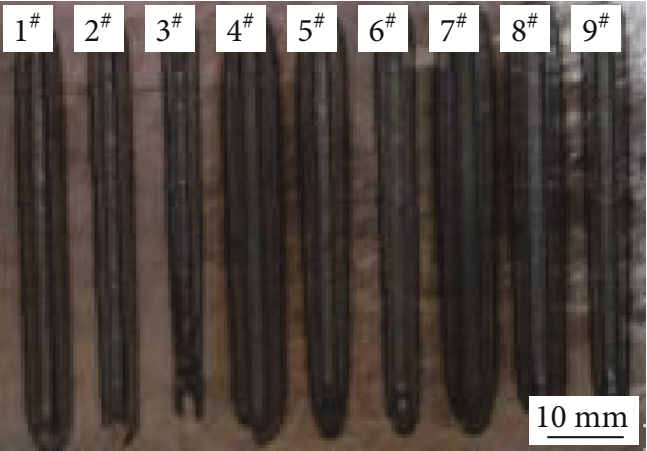
Single track samples.

It is obvious that in addition to the overlapping rate, the value of *λ* is decided by the width of one single track. Therefore, the single-track experiment needs to be made at first, measure the width of the single-track, and calculate the offset distance between adjacent tracks. Using L_9_3^4^ Taguchi OA to perform the single-track single layer LAM experiments, 9 samples with a length of 50 mm were formed (Figure 4). The width of each sample was measured, three different locations have been selected, and their averages were considered. The measured results are shown in Table 3.

**Figure 5:**
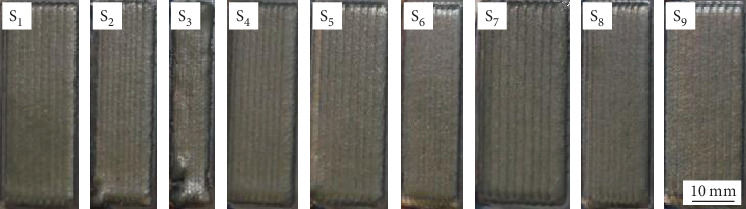
Multitrack samples.

Calculate the offset distance between adjacent tracks according to the results of single-track formation, and then using the L_9_3^4^ Taguchi OA to perform the multitrack single layer LAM experiments; the number of the track is 10; the length of every track is 50 mm; three experimental replicates were performed for better accuracy, thence the total number of samples was 27, Figure 5 shows a group of samples among them.

The quality characteristics LAM is determined by measuring surface roughness (*δ*) (Figure 3) and the width error (*w*_*e*_). The surface roughness refers to the difference height of the highest and lowest points of the sample surface. The width error refers to the difference between the theoretical calculation width (Eq. (3)) and the actual measurement width (Eq. (4)).

(3) $$\begin{array}{c} w_{c}=w+\left(t-1\right)\cdot \lambda , \end{array}$$

(4) $$\begin{array}{c} w_{e}=\left|w_{m}-w_{c}\right|, \end{array}$$

where *t* is the number of tracks (in our case *t* = 10), *w*_*c*_ is the calculated value of the sample width, and *w*_*m*_ is the measured value of the sample width. The measurement and calculation results of samples in each group were given in Table 4.

### 2.4. Microhardness and Microstructure Experiments

**Figure 6:**
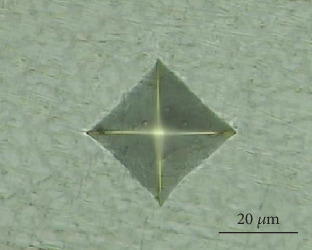
Indentation morphology.

The microstructure of the overlapping zone and the nonoverlapping zone of the cladding layer were analyzed by SEM. Microhardness tester was used to measure the transverse microhardness of the section of the cladding layer. Under the load of 4.903 N, the load was maintained for 10s, the microhardness was measured from 0.1 mm to the left of the coating, and the measured interval was about 0.1 mm, and the microhardness variation law of the overlapping zone and nonoverlapping zone of the cladding layer was obtained. The indentation morphology of measurement points was shown in Figure 6.

## 3. Results and Discussion

### 3.1. Microhardness and Microstructure Analysis

**Figure 7:**
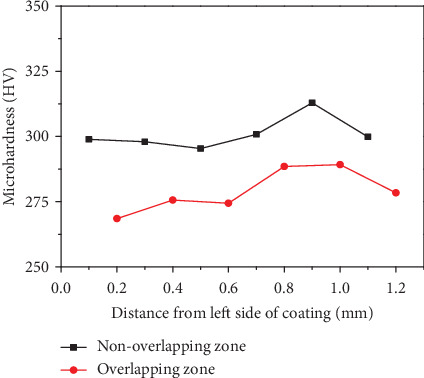
Variation of microhardness in overlapping zone and nonoverlapping zone of the cladding layer.

Through the analysis of the transverse microhardness of the cross section of the cladding layer under different process parameters, the change rule of the microhardness between overlapping zone and nonoverlapping zone was basically the same. The sample of S_6_ was taken as an example; SEM and EDS analysis were carried out on the overlapping zone and nonoverlapping zone. The transverse microhardness of cross section of the substrate and cladding layer is shown in Figure 7. The figure showed that the microhardness of the overlapping zone was lower than that of the nonoverlapping zone.

**Figure 8:**
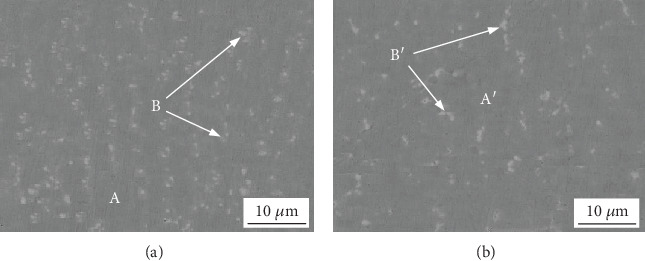
SEM images of non-overlapping zone (a) and overlapping zone (b) of coating.

The SEM images were shown in Figure 8, and the energy spectrum analysis results of A, B, A′, B′ were shown in Table 5. The SEM image of the nonoverlapping zone of the simple was shown in Figure 8(a), while the SEM image of the overlapping zone was shown in Figure 8(b). The figure shows that the coating was mainly composed of light grey matrix A and Laves phase B, and the Laves in the nonoverlapping region is dense, while Laves in the overlapping region is dispersed. This is because part of the heat input into the lap zone is absorbed by the remelting zone of the cladding layer, and the Laves phase in the lap zone is not completely precipitated due to the insufficient heat absorbed by the alloy powder, so the Laves phase content in the lap zone is lower. According to the results of energy spectrum analysis, the light gray matrix A is the solid solution of the first precipitated nickel, which is mainly composed of Ni, Cr, Mo, Nb, Fe, Si, Al, Mn, Co, and other elements. Its mass fraction is similar to that of nickel base alloy powder, which can be seen that the dilution ratio of the coating near the surface was relatively low. Both Laves phase B and matrix have high Cr content, but compared with the matrix, the content of Nb in the Laves phase significantly increased, in which Nb content was up to 24.48 wt%, and the content of Ni decreased significantly. It follows that, at this time, the Laves phase in the coating is enriched with the Nb element, and the formation of the Ni element is limited at the same time. This is because that the niobium intergranular segregation produces the Laves phase.

**Figure 9:**
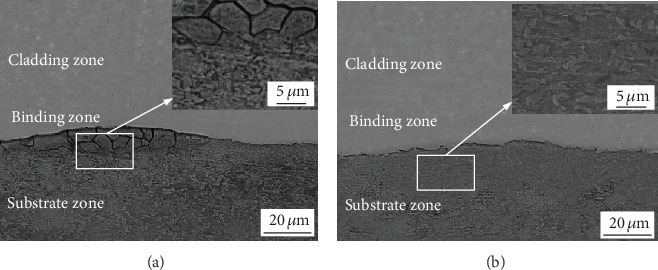
SEM images of a substrate in the overlapping zone (a) and nonoverlapping zone (b).

The SEM and partial enlarged images of the overlapping zone and nonoverlapping zone were shown in Figure 9.  Figure 9(a) shows that there are many fine cracks in the substrate of the overlapping zone compared with the substrate of the nonoverlapping zone (Figure 9(b)). It is because that the overlapping zone is subjected to the action of the double high-energy laser beam, which results in the increase of heat accumulation and temperature gradient of the substrate, the cooling shrinkage of the substrate results in a larger tensile stress, which leads to more micro cracks.

### 3.2. Signal to Noise Ratio

The change in the quality characteristics of a product under investigation, in response to the factor introduced in the experimental design, is the “signal” of the desired effect. However, when an experiment is conducted, there are numerous external factors not designed into the experiment which influence the outcome. These external factors are called the noise factors, and their effect on the outcome of the quality characteristic under test is termed “the noise”. The signal to noise ratio (S/N ratio) measures the sensitivity of the quality characteristic being investigated in a controlled manner, to those external influencing factors (noise factors) not under control. The aim of any experiment is always to determine the highest possible S/N ratio for the result. A high value of S/N implies that the signal is much higher than the random effects of the noise factors. Product design or process operation consistent with the highest S/N always yields the optimum quality with minimum variance. From the quality point of view, there are three possible categories of quality characteristics. They are (1) smaller is better, (2) nominal is best, and (3) bigger is better. The conversion of a set of observations into the S/N ratio is performed in two steps. First, the Mean Squared Deviation (MSD) of the set is calculated. Second, the S/N ratio is computed from the MSD by the equation [21, 22]:

(5) $$\begin{array}{c} \eta =-10\log_{10}\left(\text{MSD}\right). \end{array}$$

The smaller is better quality characteristic:

(6) $$\begin{array}{c} \text{MSD}=\frac{1}{n}\overset{n}{\underset{i=1}{\sum }}y_{i}^{2}. \end{array}$$

The nominal is the best quality characteristic:

(7) $$\begin{array}{c} \text{MSD}=\frac{1}{n}\overset{n}{\underset{i=1}{\sum }}{\left(y_{i}-y_{0}\right)}^{2}. \end{array}$$

The bigger is better quality characteristic:

(8) $$\begin{array}{c} \text{MSD}=\frac{1}{n}\overset{n}{\underset{i=1}{\sum }}\frac{1}{y_{i}^{2}}, \end{array}$$

where *n* is number of observations, *y*_0_ is nominal or target value.

In our case, S/N ratio was calculated for both *δ* and we considering smaller is a better criterion for both the responses by using Eq. (6). The calculated S/N ratio is shown in Table 4.

Table 6 shows the significance of parameters for surface roughness and width error. According to rang analysis, the affect order of each factor on surface roughness and width error are OR > LP > PFR > SS and OR > SS > LP > PFR, respectively.

### 3.3. Influences of Factors on Responses

**Figure 10:**
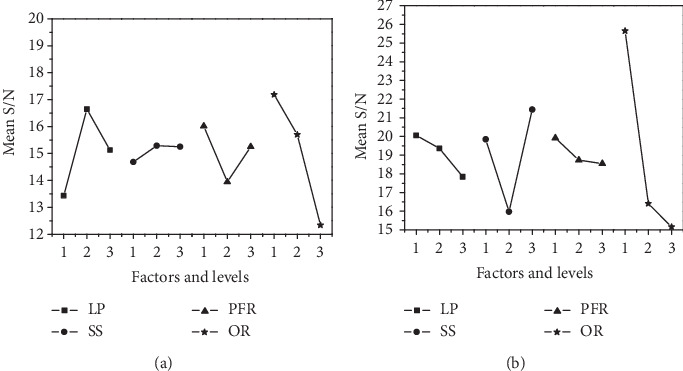
Effect of factors and their levels on mean S/N ratio of (a) surface roughness and (b) width error.

Figure 10 shows the effect of factors and their levels on the mean S/N ratio of surface roughness and width error. The highest values of S/N ratios show the levels of the factors which correspond to minimum surface roughness and width error.  Figure 10(a) shows that minimum surface roughness can be achieved at 1800 W laser power, 6 mm/s scanning speed, 10 g/min powder feed rate, and 30% overlapping rate.  Figure 10(b) shows that minimum width error can be achieved at 1600 W laser power, 8 mm/s scanning speed, 10 g/min powder feed rate, and 30% overlapping rate. Obviously, the effect of factors on surface roughness and width error is different.

### 3.4. ANOVA Analysis

Analysis of Variance (ANOVA) is routinely used to provide a measure of confidence. The technique does not directly analyze the data, but rather determines the variance of the data. By understanding the source and magnitude of variance, robust operating conditions can be predicted. Parameters used in ANOVA are calculated by the following equations [23, 24]:

(9) $$\begin{array}{c} S_{M}=\frac{{\left(\sum_{i=1}^{9}\eta_{i}\right)}^{2}}{9}, \end{array}$$

(10) $$\begin{array}{c} S_{A}=\frac{\sum_{i=1}^{3}\eta_{Ai}^{2}}{N}-S_{m}, \end{array}$$

(11) $$\begin{array}{c} S_{T}=\overset{9}{\underset{i=1}{\sum }}\eta_{i}^{2}-S_{m}, \end{array}$$

(12) $$\begin{array}{c} S_{e}=S_{T}-\sum S_{A}, \end{array}$$

(13) $$\begin{array}{c} V_{A}=\frac{S_{A}}{f_{A}}, \end{array}$$

(14) $$\begin{array}{c} F_{A}=\frac{V_{A}}{V_{e}}, \end{array}$$

where *S*_*M*_ is the average of the squares of the sums, *S*_*A*_ is the sum of squares of the control factor A (laser power, scanning speed, powder feed rate, and overlapping rate), *S*_*T*_ is the sum of squares of the variance, *S*_*e*_ is the sum of squares of the errors, *N* is the number of samples in each group (here *N* = 3), *V*_*A*_ is the variance of the control factor A, *f*_*A*_ is the degree of freedom of factor A, and *F*_*A*_ is *F*-ratio of factor A.

Table 7 shows the ANOVA for S/N ratio for surface roughness and width error. Comparing the calculated *F*-values with standard *F*-values, which can be seen that laser power, powder feeder rate, and overlapping rate have a significant effect on surface roughness at 95%, 90%, and 95% confidence levels, respectively. And for width error, it is revealed that scanning speed and overlapping rate have a significant effect at 95% and 99% confidence levels, respectively.

### 3.5. Grey Relation Analysis

Taguchi method only can be used to perform single-objective optimization, but in the present study, there are two response characteristics (surface roughness and width error) that should be considered. The present paper used Grey relation analysis (GRA) for carrying out multiobjective optimization [25, 26].

#### 3.5.1. Grey Relational Generating

In the grey relation analysis, the first step is called grey relational generating, which is to perform the normalization of measurement data to make the range between 0 and 1. According to the type of response, there are three expressions of the normalized value.

If the expectancy is higher-the-better, then the normalized value *x*_*ij*_ can be expressed as

(15) $$\begin{array}{c} x_{ij}=\frac{y_{ij}-\min y_{ij}}{\max y_{ij}-\min y_{ij}}. \end{array}$$

If the expectancy is smaller-the-better, then the normalized value *x*_*ij*_ can be expressed as

(16) $$\begin{array}{c} x_{ij}=\frac{\max y_{ij}-y_{ij}}{\max y_{ij}-\min y_{ij}}. \end{array}$$

If the expectancy is nominal-the-better, then the normalized value *x*_*ij*_ can be expressed as

(17) $$\begin{array}{c} x_{ij}=1-\frac{\left|y_{ij}-y_{0}\right|}{\max\left(\max y_{ij}-y_{0},y_{0}-\min y_{ij}\right)}. \end{array}$$

In the present research, both the surface roughness and width error are smaller-the-better type. Thence, the data in Table 4 are normalized using Eq. (16), and the normalized values are shown in Table 7.

#### 3.5.2. Grey Relational Coefficient

The grey relational coefficient can be calculated as follows:

(18) $$\begin{array}{c} \gamma \left(x_{0j},x_{ij}\right)=\frac{\Delta_{\min}+\xi \Delta_{\max}}{\Delta_{ij}+\xi \Delta_{\max}}, \end{array}$$

for *i* = 1, 2, 3, ⋯, *m*, *j* = 1, 2, 3, ⋯, *n*.

where Δ_*ij*_ = |*x*_*oj*_ − *x*_*ij*_|, *x*_0*j*_ is the reference data or best data, here, *x*_0*j*_ = 1.000. Δ_min_ = minΔ_*ij*_, Δ_max_ = maxΔ_*ij*_. *ξ* is the distinguishing coefficient, *ξ* ∈ (0, 1).

The grey relational coefficient is calculated by Eq. (18), and the results are shown in Table 7. The distinguishing coefficient is assumed at 0.5.

#### 3.5.3. Grey Relational Grade

After calculating the grey relational coefficient, the grey relational grade can be calculated by Eq. (19).

(19) $$\begin{array}{c} \Gamma \left(x_{0},x_{i}\right)=\overset{n}{\underset{j=1}{\sum }}\omega_{j}\gamma \left(x_{0j},x_{ij}\right), \end{array}$$

for *i* = 1, 2, 3, ⋯, *m*. *ω*_*j*_ is the weight of attribute *j* and usually depends on the maker's judgment or the structure of the problem, and ∑_*j*=1_^*n*^*ω*_*j*_ = 1. In the present study, the weights for the percentage change in surface roughness and width error are taken as 0.55 and 0.45, respectively. The calculated results are shown in Table 8.

#### 3.5.4. Analysis of the Grey Relational Grade

According to Table 8, the best multiple performance is achieved by experiment no. 5. That is to say, the multiobjective optimum process parametric combination is 1800 W laser power, 6 mm/s scanning speed, 30 g/min powder feeder rate, and 30% overlapping rate.  Table 9 shows the mean of grey relational grade, which indicates that the affect order of each factor on grey relational grade is OR > LP > PFR > SS.

**Figure 11:**
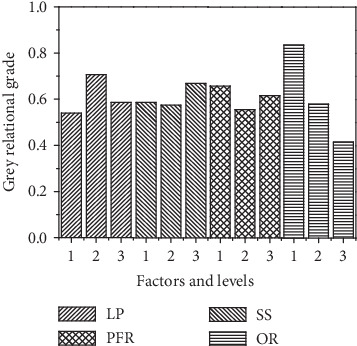
Effect of factors and their levels on grey relational grade.

**Figure 12:**
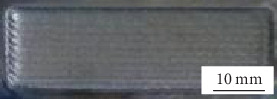
Multitrack sample of predicted parameters.

Figure 11 shows the effect of factors and their levels on grey relational grade, it can be seen; the optimal levels of laser power, scanning speed, powder feeder rate, and overlapping rate are 1800 W, 8 mm/s, 10 g/min, and 30%, respectively.  Figure 12 shows the multitrack sample with the optimum combination of parameters. The measurement results were shown in Table 10. Obviously, both the surface roughness and width error are smaller in the prediction levels than experiment no.5.

### 3.6. Regression Analysis

**Figure 13:**
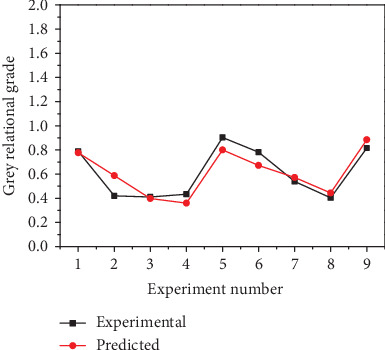
Comparison of experimental and predicted.

In the regression analysis, a mathematical model (Eq. (20)) was developed to predict the grey relational grade.  Figure 13 shows the comparison of experimental and predicted, the trends of the two curves roughly consistent.

(20) $$\begin{array}{c} \text{GRG}=1.797122+0.000115\text{LP}+0.020417\text{SS}-0.00203\text{PFR}-0.042\text{OR} \end{array}$$

## 4. Conclusions

In this paper, Inconel 625 cladding layer was prepared by LAM. The influence of major process parameters such as laser power, scanning speed, powder feed rate, and overlapping rate along with their interactions on surface roughness and width error were investigated. Taguchi method and grey relational analysis were used to optimize the selected parameters. In addition, the change of microhardness and microstructure in the overlapping zone and nonoverlapping zone of the cladding layer were explored. Through analysis, the conclusions of this study are as follows:

1. The effect of factors on surface roughness and width error is different, whereas the Taguchi method is only suitable for the optimization of a single performance characteristic. The grey relational analysis combined the entire objectives into a single value that can be used as the single characteristic in optimization issues
2. The content of Cr was high both in the Laves phase and matrix, the content of Nb in the Laves phase increases significantly and reaches up to 24.48 wt%, and the Laves in the nonoverlapping zone is more compact than overlapping zone. The microhardness of the overlapping zone of the cladding layer is lower than that of the nonoverlapping zone
3. The most significant effect in processing parameters on surface roughness and width error are both overlapping rate. The optimal levels of laser power, scanning speed, powder feeder rate, and overlapping rate are 1800 W, 8 mm/s, 10 g/min, and 30%, respectively

## Figures and Tables

**Table 1:** Powder composition of Inconel 625.

Cr	Mo	Nb	Fe	Si	C	Al	Ti	Mn	Co	Ni
21.3	8.58	3.73	0.11	0.09	0.053	0.18	0.16	0.04	0.025	Bal.

**Table 2:** Factors and levels used in Taguchi design.

Fixed factors	Value	Unit	Control factors	Symbol	Level			Unit
					1	2	3	
Standoff distance	17	mm	Laser power	LP	1600	1800	2000	W
Laser spot diameter	3	mm	Scanning speed	SS	4	6	8	mm/s
Carrier gas flow rate	450	ml/h	Powder feed rate	PFR	10	20	30	g/min
Shielding gas flow rate	300	ml/min	Overlapping rate	OR	30	35	40	%

**Table 3:** The results of single-track formation.

Expt. No.	Factors and levels			Measured values (mm)			
	LP	SS	PFR	1	2	3	Average
1	1	1	1	2.56	2.60	2.46	2.54
2	1	2	2	2.22	2.26	2.12	2.20
3	1	3	3	1.78	1.58	1.58	1.65
4	2	1	2	3.12	2.98	3.10	3.07
5	2	2	3	2.66	2.56	2.46	2.56
6	2	3	1	2.28	2.12	2.02	2.14
7	3	1	3	3.22	3.32	3.20	3.25
8	3	2	1	2.76	2.88	2.78	2.81
9	3	3	2	2.78	2.52	2.44	2.58

**Table 4:** Mean of three replicates and the S/N ratio.

Expt. No.	Factors and levels				Mean of three replicates		S/N ratio	
	LP	SS	PFR	OR	*δ* (mm)	*w*_*e*_ (mm)	*δ*	*w*_*e*_
1	1	1	1	1	0.157	0.036	16.097	28.256
2	1	2	2	2	0.223	0.164	13.146	13.948
3	1	3	3	3	0.280	0.122	11.053	17.988
4	2	1	2	3	0.241	0.130	12.391	15.872
5	2	2	3	1	0.110	0.058	19.148	22.298
6	2	3	1	2	0.120	0.083	18.396	19.900
7	3	1	3	2	0.167	0.137	15.539	15.421
8	3	2	1	3	0.210	0.214	13.549	11.658
9	3	3	2	1	0.153	0.033	16.283	26.446

**Table 5:** Results of energy dispersive spectrometer (%).

Marked location	Ni	Cr	Mo	Nb	Fe	Si	Al	Mn	Co
A	38.14	16.29	15.59	24.48	2.21	2.26	0.11	0.55	0.36
B	65.33	21.75	7.86	2.26	0.89	0.57	0.00	0.84	0.09
A′	41.71	16.47	12.03	19.46	0.71	2.30	0.00	0.54	0.21
B′	62.07	21.24	5.57	1.28	4.61	0.49	0.11	0.18	0.00

**Table 6:** Significance of factors for surface roughness and width error.

Response factor	Factors	Mean S/N ratio			Range	Rank
		Level 1	Level 2	Level 3		
Surface roughness (*δ*)	LP	13.432	16.645	15.124	3.213	2
	SS	14.676	15.281	15.244	0.605	4
	PFR	16.014	13.940	15.247	2.074	3
	OR	17.176	15.694	12.331	4.845	1

Width error (*w*_*e*_)	LP	20.064	19.357	17.842	2.222	3
	SS	19.850	15.968	21.445	5.477	2
	PFR	19.938	18.755	18.569	1.369	4
	OR	25.667	16.423	15.173	10.494	1

**Table 7:** ANOVA for S/N ratio for surface roughness and width error.

Response	Factors	*S*_*A*_	*f*_*A*_	*V*_*A*_	*F*_*A*_	% contribution
Surface roughness (*δ*)	LP	15.5	2	7.75	22.464	25.934
	SS	0.69	2	Pooled	—	—
	PFR	6.597	2	3.299	9.562	11.038
	OR	36.98	2	18.49	53.594	61.874
	Error	(0.69)	(2)	(0.345)		(1.154)
	Total	59.767	8			100

Width error (*w*_*e*_)	LP	7.735	2	Pooled	—	—
	SS	47.605	2	23.803	8.624	18.612
	PFR	3.308	2	Pooled	—	—
	OR	197.125	2	98.563	35.711	77.07
	Error	(11.043)	(4)	(2.76)		(4.318)
	Total	255.773	8			100

F_0.25_(2, 2) = 3.00; *F*_0.10_(2, 2) = 9.00; *F*_0.05_(2, 2) = 19.00; *F*_0.01_(2, 2) = 99.00; *F*_0.25_(2, 4) = 2.00; *F*_0.10_(2, 4) = 4.32; *F*_0.05_(2, 4) = 6.94; *F*_0.01_(2, 4) = 18.00.

**Table 8:** Grey relational generating, grey relational coefficient, grey relational grade, and rank for each experiment.

Expt. No.	Grey relational generating		Grey relational coefficient		Grey relational grade	Rank
	Surface roughness (*δ*)	Width error (*w*_*e*_)	Surface roughness (*δ*)	Width error (*w*_*e*_)		
1	0.724	0.983	0.644	0.967	0.789	3
2	0.335	0.276	0.429	0.408	0.420	7
3	0.000	0.508	0.333	0.504	0.410	8
4	0.229	0.464	0.393	0.485	0.434	6
5	1.000	0.862	1.000	0.784	0.903	1
6	0.941	0.724	0.894	0.644	0.782	4
7	0.665	0.425	0.599	0.465	0.539	5
8	0.412	0.000	0.460	0.333	0.403	9
9	0.747	1.000	0.664	1.000	0.815	2

**Table 9:** Influence of factors on grey relational grade.

Factors	Mean grey relational grade			Range	Rank
	Level 1	Level 2	Level 3		
LP	0.540	0.706^∗^	0.586	0.166	2
SS	0.587	0.575	0.669^∗^	0.094	4
PFR	0.658^∗^	0.556	0.617	0.102	3
OR	0.836^∗^	0.580	0.416	0.420	1

^∗^ Optimized level of parameters.

**Table 10:** Comparison between initial parameters and optimal parametric combinations.

	Initial parameters	Optimal parametric combinations	
		Prediction	Experiment
Setting level	LP_1_SS_1_PFR_1_OR_1_	LP_2_SS_3_PFR_1_OR_1_	LP_2_SS_2_PFR_3_OR_1_
Surface roughness (*δ*/mm)	0.157	0.102	0.110
Width error (*w_e_*/mm)	0.036	0.041	0.058
Grey relational grade	0.789		0.903

## Data Availability

The data used to support the findings of this study are included within the article.

## References

[1] Gonzalez J. A., Mireles J., Stafford S. W., Perez M. A., Terrazas C. A., Wicker R. B. (2019). Characterization of Inconel 625 fabricated using powder-bed-based additive manufacturing technologies. *Journal of Materials Processing Technology*.

[2] Abioye T. E., McCartney D. G., Clare A. T. (2015). Laser cladding of Inconel 625 wire for corrosion protection. *Journal of Materials Processing Technology*.

[3] Jeong G. U., Jin C. K., Seo H. Y., Kang C. G. (2019). Experimental investigation on the deformation behavior of Inconel 625 superalloy at high temperatures. *Metals*.

[4] Verdi D., Garrido M. A., Múnez C. J., Poza P. (2017). Microscale evaluation of laser cladded Inconel 625 exposed at high temperature in air. *Materials & Design*.

[5] Enrique P. D., Marzbanrad E., Mahmoodkhani Y., Jiao Z., Toyserkani E., Zhou N. Y. (2019). Surface modification of binder-jet additive manufactured Inconel 625 via electrospark deposition. *Surface & Coatings Technology*.

[6] Mostafaei A., Rodriguez de Vecchis P., Nettleship I., Chmielus M. (2019). Effect of powder size distribution on densification and microstructural evolution of binder-jet 3D-printed alloy 625. *Materials & Design*.

[7] Cui C., Uhlenwinkel V., Schulz A., Zoch H. W. (2020). Austenitic stainless steel powders with increased nitrogen content for laser additive manufacturing. *Metals*.

[8] Lai Y. B., Liu W. J., Kong Y., Wang F. Y., Zhao Y. H. (2013). Influencing factors of residual stress of Ti-6.5Al-1Mo-1V-2Zr alloy by laser rapid forming process. *Rare Metal Materials and Engineering*.

[9] Wang F., Liu W., Zhao Y., Lai Y., Han W. (2014). Subarea simulation and distributed computing of direct laser fabrication. *International Journal of Advanced Manufacturing Technology*.

[10] Liu Y., Liu C., Liu W. (2019). Optimization of parameters in laser powder deposition AlSi10Mg alloy using Taguchi method. *Optics & Laser Technology*.

[11] Yang T., Liu T., Liao W. (2019). The influence of process parameters on vertical surface roughness of the AlSi10Mg parts fabricated by selective laser melting. *Journal of Materials Processing Technology*.

[12] Liu B., Li B. Q., Li Z. (2019). Selective laser remelting of an additive layer manufacturing process on AlSi10Mg. *Results in Physics*.

[13] Yu T., Yang L., Zhao Y., Sun J., Li B. (2018). Experimental research and multi-response multi-parameter optimization of laser cladding Fe313. *Optics & Laser Technology*.

[14] Bhardwaj T., Shukla M., Paul C. P., Bindra K. S. (2019). Direct Energy Deposition - Laser Additive Manufacturing of Titanium-Molybdenum alloy: Parametric studies, microstructure and mechanical properties. *Journal of Alloys and Compounds*.

[15] Li Y., Gu D. (2014). Parametric analysis of thermal behavior during selective laser melting additive manufacturing of aluminum alloy powder. *Materials & Design*.

[16] Hu H., Ding X., Wang L. (2016). Numerical analysis of heat transfer during multi-layer selective laser melting of AlSi10Mg. *Optik*.

[17] Conti P., Cianetti F., Pilerci P. (2018). Parametric finite elements model of SLM additive manufacturing process. *Procedia Structural Integrity*.

[18] Pleass C., Jothi S. (2018). Influence of powder characteristics and additive manufacturing process parameters on the microstructure and mechanical behaviour of Inconel 625 fabricated by selective laser melting. *Additive Manufacturing*.

[19] Yang W. H., Tarng Y. S. (1998). Design optimization of cutting parameters for turning operations based on the Taguchi method. *Journal of Materials Processing Technology*.

[20] Lai Y. B., Zhang B. H., Zhao J. B., Liu W. J., Zhao Y. H. (2016). Calculation and experimental verification of optimal overlapping ratio in laser metal direct manufacturing. *Transactions of the China Welding Institution*.

[21] Balaram Naik A., Chennakeshava Reddy A. (2018). Optimization of tensile strength in TIG welding using the Taguchi method and analysis of variance (ANOVA). *Thermal Science and Engineering Progress*.

[22] Bademlioglu A. H., Canbolat A. S., Yamankaradeniz N., Kaynakli O. (2018). Investigation of parameters affecting Organic Rankine Cycle efficiency by using Taguchi and ANOVA methods. *Applied Thermal Engineering*.

[23] Viswanath Allamraju K. (2018). Voltage optimization of piezoelectric circular transducer by Taguchi and ANOVA approches. *Materials Today: Proceedings*.

[24] Dongxia Y., Xiaoyan L., Dingyong H., Zuoren N., Hui H. (2012). Optimization of weld bead geometry in laser welding with filler wire process using Taguchi's approach. *Optics and Laser Technology*.

[25] Kanchana J., Prasath V., Krishnaraj V., Geetha Priyadharshini B. (2019). Multi response optimization of process parameters using grey relational analysis for milling of hardened custom 465 steel. *Procedia Manufacturing*.

[26] Equbal M. I., kumar R., Shamim M., Ohdar R. K. (2014). A grey-based Taguchi method to optimize hot forging process. *Procedia Materials Science*.

